# 2-Hy­droxy­ethanaminium 3,4,5,6-tetra­bromo-2-(meth­oxy­carbon­yl)benzoate methanol monosolvate

**DOI:** 10.1107/S160053681100852X

**Published:** 2011-03-12

**Authors:** Jian Li

**Affiliations:** a Department of Chemistry and Chemical Engineering, Weifang University, Weifang 261061, People’s Republic of China

## Abstract

In the title compound, C_2_H_8_NO^+^·C_9_H_3_Br_4_O_4_^−^·CH_4_O, inter­molecular N—H⋯O and O—H⋯O hydrogen bonds link the components into chains along [001].

## Related literature

For related structures, see: Li (2011 ▶); Liang (2008 ▶). 
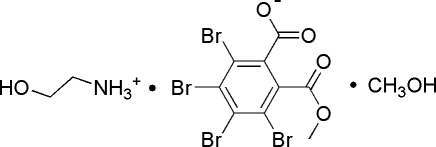


## Experimental

### 

#### Crystal data

C_2_H_8_NO^+^·C_9_H_3_Br_4_O_4_^−^·CH_4_O
*M* *_r_* = 588.89
Monoclinic, 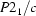

*a* = 9.4231 (11) Å
*b* = 25.475 (2) Å
*c* = 8.3463 (7) Å
β = 111.990 (1)°
*V* = 1857.8 (3) Å^3^
*Z* = 4
Mo *K*α radiation
μ = 8.69 mm^−1^
*T* = 298 K
0.42 × 0.35 × 0.34 mm

#### Data collection

Bruker SMART CCD diffractometer
Absorption correction: multi-scan (*SADABS*; Bruker, 1997 ▶) *T*_min_ = 0.121, *T*_max_ = 0.156
9367 measured reflections
3269 independent reflections
1581 reflections with *I* > 2σ(*I*)
*R*_int_ = 0.086

#### Refinement

*R*[*F* ^2^ > 2σ(*F* ^2^)] = 0.044
*wR*(*F* ^2^) = 0.071
*S* = 1.00
3269 reflections
212 parameters
H-atom parameters constrained
Δρ_max_ = 0.55 e Å^−3^
Δρ_min_ = −0.58 e Å^−3^

### 

Data collection: *SMART* (Bruker, 1997 ▶); cell refinement: *SAINT* (Bruker, 1997 ▶); data reduction: *SAINT*; program(s) used to solve structure: *SHELXS97* (Sheldrick, 2008 ▶); program(s) used to refine structure: *SHELXL97* (Sheldrick, 2008 ▶); molecular graphics: *SHELXTL* (Sheldrick, 2008 ▶) and *PLATON* (Spek, 2009 ▶); software used to prepare material for publication: *SHELXTL*.

## Supplementary Material

Crystal structure: contains datablocks global, I. DOI: 10.1107/S160053681100852X/kj2169sup1.cif

Structure factors: contains datablocks I. DOI: 10.1107/S160053681100852X/kj2169Isup2.hkl

Additional supplementary materials:  crystallographic information; 3D view; checkCIF report

## Figures and Tables

**Table 1:** Hydrogen-bond geometry (Å, °)

*D*—H⋯*A*	*D*—H	H⋯*A*	*D*⋯*A*	*D*—H⋯*A*
N1—H1*A*⋯O3^i^	0.89	2.01	2.885 (7)	168
N1—H1*B*⋯O6	0.89	1.86	2.740 (7)	168
N1—H1*C*⋯O4^ii^	0.89	1.96	2.789 (8)	154
O5—H5⋯O4^ii^	0.82	2.00	2.813 (8)	169
O6—H6⋯O3^iii^	0.82	1.92	2.714 (8)	163

Symmetry codes: (i) 
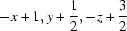
; (ii) 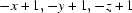; (iii) 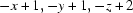.

**Fig. 1.**
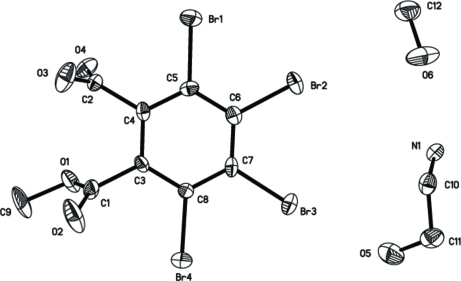
The molecular structure of the title compound, drawn with 30% probability ellipsoids.

**Fig. 2.**
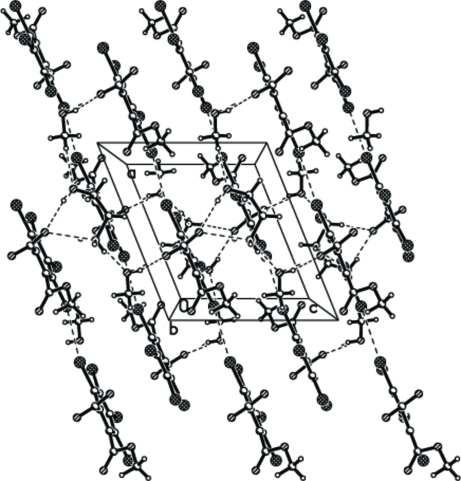
The crystal packing of the title compound, viewed along the b axis. Hydrogen bonds are indicated by dashed lines.

## supplementary crystallographic information

### Comment

4,5,6,7-Tetrabromo-2-ethylisoindoline-1,3-dione is an important flame retardant. 2-(Methoxycarbonyl)-3,4,5,6-tetrabromobenzoic acid is the intermediate in the synthesis of 4,5,6,7-Tetrabromo-2-ethylisoindoline-1,3-dione. In this paper, the structure of the title compound is reported. The asymmetric unit of the title compound (I) contains one ethanolaminium cation, one 2-(methoxycarbonyl)-3,4,5,6-tetrabromobenzoate anion and one methanol solvent molecule (Fig. 1). The bond lengths and angles agree with those in ethanaminium 2-(methoxycarbonyl)-3,4,5,6-tetrabromo benzoate methanol solvate (Li, 2011) and ethane-1,2-diaminium 2-(methoxycarbonyl)-3,4,5,6-tetrabromo benzoate methanol solvate (Liang, 2008). In the crystal structure, intermolecular N—H···O and O—H···O hydrogen bonds link the components of the structure into one-dimensional chains along [001](see Fig. 2 and Table 1).

### Experimental

A mixture of 4,5,6,7-tetrabromoisobenzofuran-1,3-dione (4.64 g, 0.01 mol) and methanol (15 ml) was refluxed for 0.5 h. Ethanolamine (0.61 g, 0.01 mol) was added to this solution, followed by stirring for 10 min at room temperature. The solution was kept at room temperature for 5 d. Natural evaporation gave colourless single crystals of the title compound, suitable for X-ray analysis.

### Refinement

H atoms were initially located from difference maps and then refined in a riding model with C—H = 0.96–0.97 Å, N—H = 0.89 Å, O—H = 0.82Å and *U*_iso_(H) = 1.2*U*_eq_(C) or 1.5*U*_eq_(O, N, methyl C).

### Crystal data

**Table:**

C_2_H_8_NO^+^·C_9_H_3_Br_4_O_4_^−^·CH_4_O	*F*(000) = 1128
*M**_r_* = 588.89	*D*_x_ = 2.105 Mg m^−^^3^
Monoclinic, *P*2_1_/*c*	Mo *K*α radiation, λ = 0.71073 Å
*a* = 9.4231 (11) Å	Cell parameters from 1705 reflections
*b* = 25.475 (2) Å	θ = 2.9–21.8°
*c* = 8.3463 (7) Å	µ = 8.69 mm^−^^1^
β = 111.990 (1)°	*T* = 298 K
*V* = 1857.8 (3) Å^3^	Block, colorless
*Z* = 4	0.42 × 0.35 × 0.34 mm

### Data collection

**Table:**

Bruker SMART CCD diffractometer	3269 independent reflections
Radiation source: fine-focus sealed tube	1581 reflections with *I* > 2σ(*I*)
graphite	*R*_int_ = 0.086
φ and ω scans	θ_max_ = 25.0°, θ_min_ = 2.5°
Absorption correction: multi-scan (*SADABS*; Bruker, 1997)	*h* = −10→11
*T*_min_ = 0.121, *T*_max_ = 0.156	*k* = −30→28
9367 measured reflections	*l* = −9→9

### Refinement

**Table:**

Refinement on *F*^2^	Secondary atom site location: difference Fourier map
Least-squares matrix: full	Hydrogen site location: inferred from neighbouring sites
*R*[*F*^2^ > 2σ(*F*^2^)] = 0.044	H-atom parameters constrained
*wR*(*F*^2^) = 0.071	*w* = 1/[σ^2^(*F*_o_^2^) + (0.0103*P*)^2^] where *P* = (*F*_o_^2^ + 2*F*_c_^2^)/3
*S* = 1.00	(Δ/σ)_max_ = 0.001
3269 reflections	Δρ_max_ = 0.55 e Å^−^^3^
212 parameters	Δρ_min_ = −0.58 e Å^−^^3^
0 restraints	Extinction correction: *SHELXL97* (Sheldrick, 2008), Fc^*^=kFc[1+0.001xFc^2^λ^3^/sin(2θ)]^-1/4^
Primary atom site location: structure-invariant direct methods	Extinction coefficient: 0.00077 (5)

### Special details

**Table:**

Geometry. All e.s.d.'s (except the e.s.d. in the dihedral angle between two l.s. planes) are estimated using the full covariance matrix. The cell e.s.d.'s are taken into account individually in the estimation of e.s.d.'s in distances, angles and torsion angles; correlations between e.s.d.'s in cell parameters are only used when they are defined by crystal symmetry. An approximate (isotropic) treatment of cell e.s.d.'s is used for estimating e.s.d.'s involving l.s. planes.
Refinement. Refinement of *F*^2^ against ALL reflections. The weighted *R*-factor *wR* and goodness of fit *S* are based on *F*^2^, conventional *R*-factors *R* are based on *F*, with *F* set to zero for negative *F*^2^. The threshold expression of *F*^2^ > σ(*F*^2^) is used only for calculating *R*-factors(gt) *etc*. and is not relevant to the choice of reflections for refinement. *R*-factors based on *F*^2^ are statistically about twice as large as those based on *F*, and *R*- factors based on ALL data will be even larger.

### Fractional atomic coordinates and isotropic or equivalent isotropic displacement parameters (Å^2^)

**Table:**

	*x*	*y*	*z*	*U*_iso_*/*U*_eq_	
Br1	0.33226 (9)	0.40176 (3)	0.76997 (13)	0.0614 (3)	
Br2	0.40915 (10)	0.52775 (3)	0.75108 (12)	0.0620 (3)	
Br3	0.73518 (10)	0.56240 (3)	0.72387 (12)	0.0632 (3)	
Br4	0.98636 (10)	0.47177 (3)	0.73227 (13)	0.0709 (3)	
N1	0.4471 (6)	0.6959 (2)	0.6870 (8)	0.0456 (18)	
H1A	0.4218	0.7297	0.6773	0.068*	
H1B	0.3755	0.6775	0.7077	0.068*	
H1C	0.4542	0.6848	0.5892	0.068*	
O1	0.8414 (6)	0.3298 (2)	0.6314 (8)	0.0643 (18)	
O2	1.0002 (7)	0.3545 (2)	0.8847 (9)	0.092 (2)	
O3	0.6507 (6)	0.30316 (19)	0.8975 (8)	0.0702 (18)	
O4	0.5049 (6)	0.30677 (18)	0.6230 (8)	0.0567 (17)	
O5	0.7644 (6)	0.6848 (2)	0.6739 (8)	0.081 (2)	
H5	0.6920	0.6850	0.5807	0.097*	
O6	0.2414 (7)	0.6457 (2)	0.7949 (8)	0.095 (2)	
H6	0.2570	0.6592	0.8891	0.114*	
C1	0.8828 (10)	0.3586 (3)	0.7696 (14)	0.051 (2)	
C2	0.5883 (10)	0.3264 (3)	0.7595 (14)	0.044 (2)	
C3	0.7674 (8)	0.4002 (2)	0.7569 (9)	0.0337 (19)	
C4	0.6248 (8)	0.3843 (3)	0.7589 (9)	0.0342 (19)	
C5	0.5202 (7)	0.4236 (3)	0.7578 (9)	0.038 (2)	
C6	0.5502 (8)	0.4761 (2)	0.7492 (9)	0.037 (2)	
C7	0.6908 (9)	0.4915 (2)	0.7421 (10)	0.041 (2)	
C8	0.7971 (8)	0.4530 (2)	0.7450 (10)	0.038 (2)	
C9	0.9453 (9)	0.2862 (3)	0.6319 (12)	0.096 (3)	
H9A	1.0227	0.2988	0.5928	0.145*	
H9B	0.8878	0.2588	0.5563	0.145*	
H9C	0.9927	0.2726	0.7471	0.145*	
C10	0.5970 (9)	0.6887 (3)	0.8323 (10)	0.052 (2)	
H10A	0.5915	0.7038	0.9365	0.062*	
H10B	0.6174	0.6514	0.8525	0.062*	
C11	0.7246 (9)	0.7134 (3)	0.7971 (11)	0.061 (3)	
H11A	0.6954	0.7488	0.7547	0.074*	
H11B	0.8133	0.7158	0.9040	0.074*	
C12	0.1062 (10)	0.6179 (3)	0.7424 (12)	0.087 (3)	
H12A	0.1093	0.5932	0.8306	0.130*	
H12B	0.0927	0.5994	0.6375	0.130*	
H12C	0.0224	0.6417	0.7225	0.130*	

### Atomic displacement parameters (Å^2^)

**Table:**

	*U*^11^	*U*^22^	*U*^33^	*U*^12^	*U*^13^	*U*^23^
Br1	0.0395 (5)	0.0646 (5)	0.0851 (8)	−0.0002 (5)	0.0292 (5)	0.0080 (6)
Br2	0.0537 (6)	0.0497 (5)	0.0860 (8)	0.0231 (5)	0.0299 (6)	0.0052 (5)
Br3	0.0653 (6)	0.0307 (4)	0.0958 (9)	−0.0021 (5)	0.0328 (6)	0.0015 (5)
Br4	0.0410 (6)	0.0579 (5)	0.1211 (10)	−0.0072 (5)	0.0387 (6)	−0.0055 (6)
N1	0.050 (5)	0.036 (3)	0.052 (5)	−0.013 (3)	0.020 (4)	−0.010 (4)
O1	0.044 (4)	0.061 (4)	0.078 (5)	0.021 (3)	0.012 (4)	−0.019 (4)
O2	0.060 (5)	0.086 (4)	0.092 (6)	0.041 (4)	−0.016 (4)	−0.030 (5)
O3	0.097 (5)	0.041 (3)	0.050 (5)	−0.003 (3)	0.001 (4)	0.011 (3)
O4	0.062 (4)	0.048 (3)	0.053 (5)	−0.022 (3)	0.014 (4)	−0.006 (3)
O5	0.049 (4)	0.097 (5)	0.093 (6)	0.017 (4)	0.023 (4)	0.017 (4)
O6	0.082 (5)	0.139 (5)	0.076 (5)	−0.057 (5)	0.042 (4)	−0.023 (4)
C1	0.037 (6)	0.037 (5)	0.064 (8)	−0.002 (5)	0.002 (6)	−0.010 (5)
C2	0.035 (6)	0.030 (5)	0.066 (8)	−0.003 (4)	0.018 (6)	−0.004 (5)
C3	0.021 (4)	0.031 (4)	0.046 (6)	0.008 (4)	0.008 (4)	−0.001 (4)
C4	0.031 (5)	0.035 (4)	0.031 (6)	0.006 (4)	0.004 (4)	0.003 (4)
C5	0.027 (5)	0.045 (4)	0.042 (6)	−0.005 (4)	0.012 (4)	0.001 (4)
C6	0.038 (5)	0.025 (4)	0.048 (6)	0.004 (4)	0.014 (4)	−0.002 (4)
C7	0.044 (5)	0.025 (4)	0.053 (6)	0.006 (4)	0.019 (5)	0.010 (4)
C8	0.025 (5)	0.038 (4)	0.044 (6)	−0.001 (4)	0.005 (4)	−0.002 (4)
C9	0.078 (7)	0.082 (7)	0.114 (9)	0.044 (6)	0.019 (7)	−0.041 (6)
C10	0.067 (7)	0.033 (4)	0.052 (7)	0.010 (5)	0.019 (6)	0.005 (5)
C11	0.039 (6)	0.066 (6)	0.070 (8)	0.006 (5)	0.010 (5)	0.004 (5)
C12	0.067 (7)	0.092 (7)	0.113 (10)	−0.023 (6)	0.047 (7)	−0.027 (7)

### Geometric parameters (Å, °)

**Table:**

Br1—C5	1.894 (6)	C2—C4	1.514 (9)
Br2—C6	1.875 (6)	C3—C8	1.384 (8)
Br3—C7	1.872 (6)	C3—C4	1.410 (8)
Br4—C8	1.888 (6)	C4—C5	1.402 (8)
N1—C10	1.489 (8)	C5—C6	1.376 (8)
N1—H1A	0.8900	C6—C7	1.404 (8)
N1—H1B	0.8900	C7—C8	1.397 (8)
N1—H1C	0.8900	C9—H9A	0.9600
O1—C1	1.298 (9)	C9—H9B	0.9600
O1—C9	1.480 (7)	C9—H9C	0.9600
O2—C1	1.166 (9)	C10—C11	1.481 (8)
O3—C2	1.232 (9)	C10—H10A	0.9700
O4—C2	1.223 (9)	C10—H10B	0.9700
O5—C11	1.420 (8)	C11—H11A	0.9700
O5—H5	0.8200	C11—H11B	0.9700
O6—C12	1.377 (8)	C12—H12A	0.9600
O6—H6	0.8200	C12—H12B	0.9600
C1—C3	1.493 (9)	C12—H12C	0.9600
			
C10—N1—H1A	109.5	C6—C7—Br3	121.1 (5)
C10—N1—H1B	109.5	C3—C8—C7	121.5 (6)
H1A—N1—H1B	109.5	C3—C8—Br4	118.1 (5)
C10—N1—H1C	109.5	C7—C8—Br4	120.5 (5)
H1A—N1—H1C	109.5	O1—C9—H9A	109.5
H1B—N1—H1C	109.5	O1—C9—H9B	109.5
C1—O1—C9	116.4 (6)	H9A—C9—H9B	109.5
C11—O5—H5	109.5	O1—C9—H9C	109.5
C12—O6—H6	109.5	H9A—C9—H9C	109.5
O2—C1—O1	123.9 (8)	H9B—C9—H9C	109.5
O2—C1—C3	124.3 (9)	C11—C10—N1	112.3 (6)
O1—C1—C3	111.7 (8)	C11—C10—H10A	109.2
O4—C2—O3	126.1 (7)	N1—C10—H10A	109.2
O4—C2—C4	117.6 (9)	C11—C10—H10B	109.2
O3—C2—C4	116.2 (8)	N1—C10—H10B	109.2
C8—C3—C4	119.9 (6)	H10A—C10—H10B	107.9
C8—C3—C1	122.2 (6)	O5—C11—C10	112.3 (6)
C4—C3—C1	117.9 (6)	O5—C11—H11A	109.1
C5—C4—C3	117.8 (6)	C10—C11—H11A	109.1
C5—C4—C2	122.3 (6)	O5—C11—H11B	109.1
C3—C4—C2	119.9 (6)	C10—C11—H11B	109.1
C6—C5—C4	122.4 (6)	H11A—C11—H11B	107.9
C6—C5—Br1	120.2 (5)	O6—C12—H12A	109.5
C4—C5—Br1	117.4 (5)	O6—C12—H12B	109.5
C5—C6—C7	119.4 (6)	H12A—C12—H12B	109.5
C5—C6—Br2	121.5 (5)	O6—C12—H12C	109.5
C7—C6—Br2	119.2 (5)	H12A—C12—H12C	109.5
C8—C7—C6	119.0 (5)	H12B—C12—H12C	109.5
C8—C7—Br3	119.9 (5)		
			
C9—O1—C1—O2	6.2 (13)	C4—C5—C6—C7	−0.4 (11)
C9—O1—C1—C3	−178.2 (6)	Br1—C5—C6—C7	179.3 (5)
O2—C1—C3—C8	63.9 (13)	C4—C5—C6—Br2	−179.1 (5)
O1—C1—C3—C8	−111.7 (8)	Br1—C5—C6—Br2	0.5 (9)
O2—C1—C3—C4	−115.6 (10)	C5—C6—C7—C8	−0.4 (11)
O1—C1—C3—C4	68.9 (9)	Br2—C6—C7—C8	178.4 (6)
C8—C3—C4—C5	−3.3 (11)	C5—C6—C7—Br3	178.2 (6)
C1—C3—C4—C5	176.1 (7)	Br2—C6—C7—Br3	−3.0 (9)
C8—C3—C4—C2	175.8 (8)	C4—C3—C8—C7	2.7 (12)
C1—C3—C4—C2	−4.7 (11)	C1—C3—C8—C7	−176.7 (7)
O4—C2—C4—C5	77.2 (10)	C4—C3—C8—Br4	−177.1 (5)
O3—C2—C4—C5	−105.4 (9)	C1—C3—C8—Br4	3.4 (11)
O4—C2—C4—C3	−101.9 (9)	C6—C7—C8—C3	−0.8 (12)
O3—C2—C4—C3	75.5 (10)	Br3—C7—C8—C3	−179.5 (6)
C3—C4—C5—C6	2.2 (11)	C6—C7—C8—Br4	179.0 (5)
C2—C4—C5—C6	−176.9 (8)	Br3—C7—C8—Br4	0.4 (9)
C3—C4—C5—Br1	−177.5 (5)	N1—C10—C11—O5	−73.5 (8)
C2—C4—C5—Br1	3.4 (10)		

### Hydrogen-bond geometry (Å, °)

**Table:**

*D*—H···*A*	*D*—H	H···*A*	*D*···*A*	*D*—H···*A*
N1—H1A···O3^i^	0.89	2.01	2.885 (7)	168
N1—H1B···O6	0.89	1.86	2.740 (7)	168
N1—H1C···O4^ii^	0.89	1.96	2.789 (8)	154
O5—H5···O4^ii^	0.82	2.00	2.813 (8)	169
O6—H6···O3^iii^	0.82	1.92	2.714 (8)	163

Symmetry codes: (i) −*x*+1, *y*+1/2, −*z*+3/2; (ii) −*x*+1, −*y*+1, −*z*+1; (iii) −*x*+1, −*y*+1, −*z*+2.
